# Cryptomenorrhea Due to Imperforate Hymen Leading to a Massive Hematocolpos

**DOI:** 10.7759/cureus.29038

**Published:** 2022-09-11

**Authors:** Mukta Agarwal, Sudwita Sinha, Upasna Sinha, Simran Dureja, Ishita Roy

**Affiliations:** 1 Obstetrics and Gynecology, All India Institute of Medical Sciences, Patna, IND; 2 Radiology, All India Institute of Medical Sciences, Patna, IND

**Keywords:** hymenotomy, cruciate incision, 3.4 liters hematocolpos, imperforate hymen, massive hematocolpos, cryptomenorrhea

## Abstract

Herein, we present a case of cryptomenorrhea due to imperforate hymen where approximately 3400 ml of retained menstrual blood was drained in a 16-year-old girl who presented with primary amenorrhea, cyclical abdominal pain, mass abdomen and acute pain abdomen for 15 days. Magnetic resonance imaging revealed hematocolpos due to imperforate hymen with grossly distended vagina measuring 28.6 × 9.9 × 11.3 cm. Surgical drainage of hematocolpos was done by performing hymenotomy with a cruciate-shaped incision. The post-operative period was uneventful and patient was discharged the next day in stable condition. To our knowledge, this is the first case of hematocolpos reported in literature where more than 3 liters of retained menstrual blood was drained.

## Introduction

Imperforate hymen is one of the obstructive congenital anomalies of the female genital tract which is rare with a prevalence of 0.05-0.1% [1]. It was first described by Ambroise Pare in 1633 [2]. Although retention of menses was known in ancient times, there are vague and incomplete descriptions [3]. Rhazes, an Arab physician (923 A.D.), recognized imperforate hymen for which he advised incision followed by daily coitus [3]. Hildanus (1560-1634 A.D.) described three cases of imperforate hymen [3]. The hymen is an embryological remnant of mesodermal tissue which is supposed to normally perforate during the later stages of embryo development [4]. Absence of perforation of this membrane is called imperforate hymen [4]. It results due to complete failure of canalisation of the inferior end of the vaginal plate between the sinovaginal bulb of the vagina and urogenital sinus [1]. This hampers outflow of menstrual blood leading to its accumulation in the vagina or the uterus [1]. It commonly presents around puberty or during the newborn period and childhood [1]. Treatment is simple hymenotomy with proper incisions on imperforate hymenal membrane to provide an annular intact hymen [1].

## Case presentation

We present here a case of a 16-year-old girl who presented in the outpatient department of Obstetrics and Gynaecology in All India Institute of Medical Sciences, Patna, with chief complaints of not attaining menses yet, cyclical pain abdomen lasting for four to five days coming at an interval of around one month and acute pain abdomen for 15 days. The pain abdomen was not associated with nausea, vomiting or diarrhoea. There was no history of altered bowel habits or urinary complaints. There was no significant past medical or surgical history. On examination patient was alert, conscious, oriented and co-operative. She was of average built and fairly nourished. Her vitals were normal and she was afebrile to touch. Systemic examination was normal. Her secondary sexual characters were well developed with breast and pubic hair tanner stage 4. Abdominal examination revealed a lower mid-abdominal mass about the size of a 32-week pregnancy. The mass was slightly tender, dull to percussion, and transmitted a fluid thrill.

**Figure 1:**
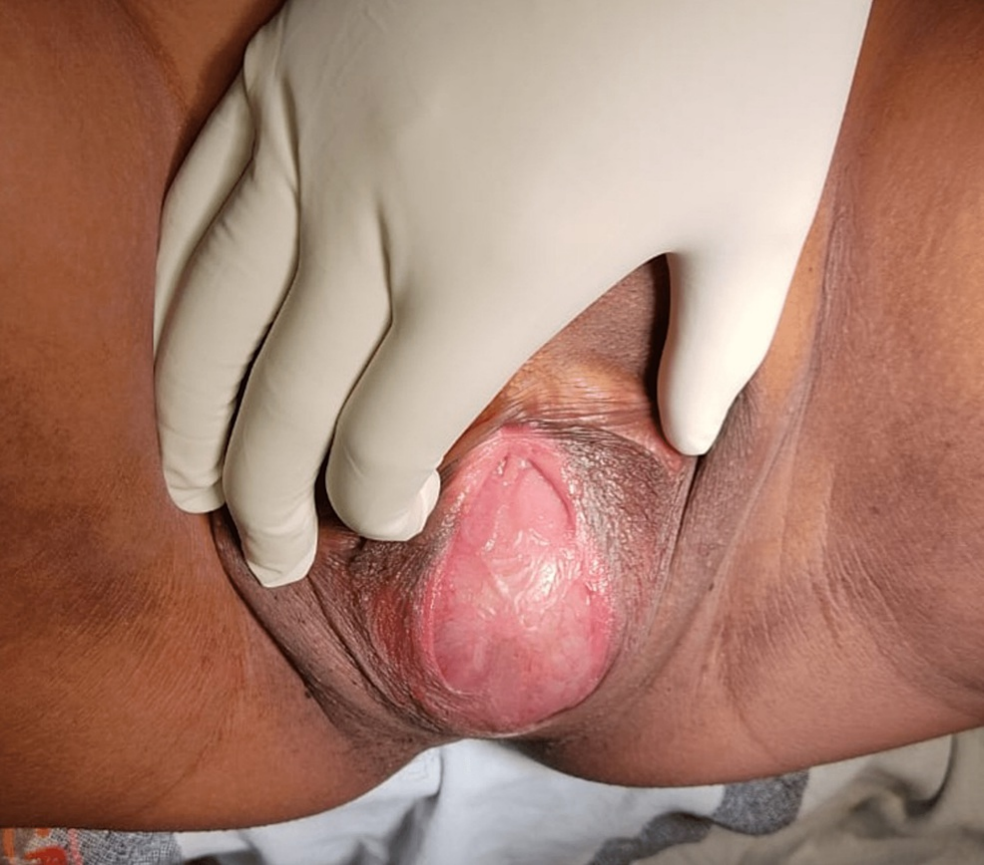
The perineum showing imperforate hymen

Examination of the external genitalia revealed bulging of the entire perineum. The hymen was intact. There was a 6×6 cm bulging membrane posterior to the urethra, which was non-tender, but was exaggerated on doing Valsalva manoeuvre. Labia majora was normal whereas the labia minora was fused with the bulging membrane. Figure 1 shows the image of the bulging perineum due to imperforate hymen with massive hematocolpos.

**Figure 2:**
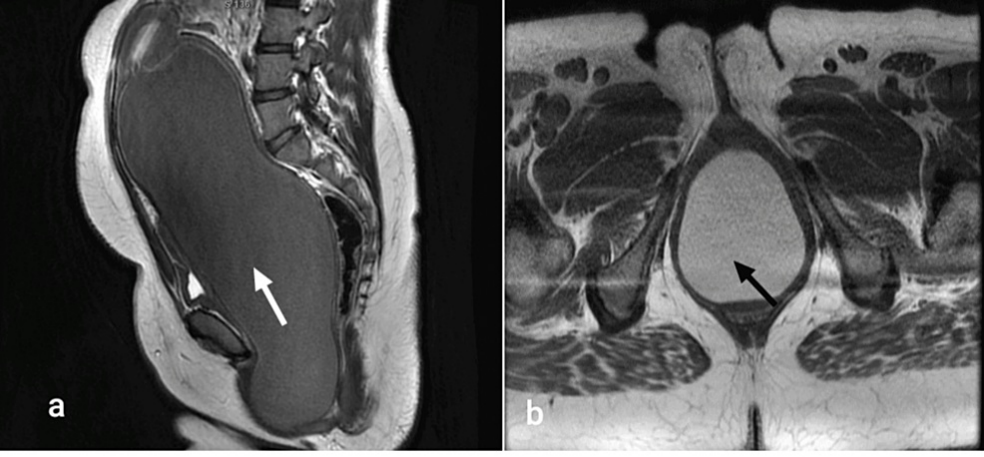
(a) Sagittal T2 weighted image and (b) Axial T1 weighted image showsT2 hypointense (white arrow) and T1 hyperintense (black arrow) collection in the grossly distended vagina till the level of hymen suggestive of hematocolpos

A provisional diagnosis of imperforate hymen with hematocolpos was made. Magnetic resonance imaging (MRI) was done to confirm hematocolpos and to look for associated hematometra, hematosalpinx and any other associated congenital anomalies of the genito-urinary tract which revealed a grossly distended vagina measuring 28.6 ×9.9 × 11.3 cm (cranio-caudal × antero-posterior × transverse) till the level of hymen, filled with T1 hyperintense and T2 hypointense fluid collections suggestive of hemorrhagic collection in vagina consistent with hematocolpos. The uterus measured 5.8 × 5.2 × 3.2 cm, was displaced superiorly and was normal. Superiorly it was reaching up to L3-L4 inter-vertebral disc level. Anteriorly it was abutting the anterior abdominal wall, compressing the urinary bladder and posteriorly it was compressing the rectum and recto-sigmoid. Bilateral ovaries appeared normal and were displaced supero-laterally. A diagnosis of massive hematocolpos with imperforate hymen was confirmed. Figure 2 shows the MRI images of massive hematocolpos.

Patient was taken up for surgery after taking written informed consent. Hymenotomy was performed under general anaesthesia after catheterization of the urethra. A stab incision was first made in the centre of the imperforate hymen which was then extended in a cruciate manner. The hymen was thick and tough. Around 3400 ml of thick tarry coloured old menstrual blood were drained. Redundant flaps were cut and the edges of the margins were everted by suturing the inner vaginal mucosa to the exterior vestibular mucosa. The abdominal mass disappeared and the enlargement of the abdomen subsided as the vagina emptied. Culture of the vaginal fluid showed no growth. Antibiotics were prescribed and perineal hygiene was maintained. Catheter was removed as soon as the patient could ambulate. She had an uneventful post-operative period and was discharged the next day in stable and satisfactory condition. On follow-up after six weeks, patient started having normal flow of her menses.

## Discussion

Imperforate hymen with hematocolpos or hematometra is a slightly rare and well-known congenital anomaly of the female genital tract by most pediatric and gynecologic surgeons, which usually occurs isolated but can vary rarely be associated with other female genitourinary tract anomalies or genetic disorders [1]. Hence, other associated Mullerian anomalies should be ruled out. Although it is sporadic in occurrence, multiple familial cases have been reported with both dominant and recessive inheritance [1]. During fetal life, the membranous structure at the junction of urogenital sinus and sinovaginal bulb known as hymen becomes patent to establish normal outflow tract [1]. Hymen is a thin membrane of stratified squamous epithelium which circumscribes the vaginal introitus [5]. When the hymen does not spontaneously rupture during neonatal development, it is referred to as an imperforate hymen. Hymen provides a physical barrier to infection before puberty when immunity of vagina is not yet fully developed [1]. Imperforate hymen can be easily diagnosed with complete history and clinical examination. Patients are usually asymptomatic until onset of menarche after which menstrual blood begins to accumulate in the vagina leading to a spectrum of symptoms constituting hematocolpos, hematometra and hematosalpinx resulting in symptoms such as primary or secondary amenorrhea, recurrent cyclical lower abdominal pain, bluish bulge at the vaginal introitus, acute or chronic retention of urine, mass per abdomen, endometriosis due to retrograde menstruation or rarely constipation and intestinal obstruction [1]. In our case, patient presented with primary amenorrhea, mass abdomen and cyclical abdominal pain. Other disorders of female outflow tract (transverse vaginal septum), appendicitis, nephrolithiasis and abdominal tumor constitute the differential diagnoses. Treatment is surgical drainage of hematocolpos after performing hymenotomy avoiding injury to the urethra and Bartholin’s glands. To achieve this, incisions are avoided at 5 and 7 o’clock positions to respect the orifices of the Bartholin’s glands and incisions are made at 11 o’clock position to free the lower bank of urinary meatus and ensure meato-hymenal dissociation [6]. It is important to maintain asepsis during surgery since a closed vagina lacks the protective Doderlein’s bacilli resulting in alkaline or weakly acidic pH of vagina and poor natural resistance to bacteria entering from lower genital tract in addition to the fact that blood and debris provide a good culture medium for bacterial growth [4]. There are various incisions described for hymenotomy in imperforate hymen. The most common cruciate or cruciform incision consists of incising the hymenal membrane in a cross-shaped fashion [2]. Graber’s radial incision begins with a central hymenectomy followed by radial incisions of the hymenal ring [2]. Caparo’s incision, the only technique for retaining virginity, is a simple technique of allowing a sagittal incision on the hymen giving a lipped hymen [2]. Elliptical excision is another technique of hymenotomy [2]. Pozzi technique consists of transversely incising the hymen and the constrictor muscles of the vulva at 5 and 7 o’clock positions and suturing circularly at several points [2]. In carbon dioxide laser technique, a mixture of carbon dioxide, nitrogen and helium gas is used like a scalpel [2]. Complications of hymenotomy include bleeding, scarring and restenosis of vaginal opening [2]. Recurrence is rare.

Although numerous cases of imperforate hymen with hematocolpos drainage have been reported in literature, the total volume of tarry-colored menstrual blood drained is usually more or less around one liter. After a thorough review of literature, we found one such case wherein Cecutti (1964) reported a case of imperforate hymen with hematocolpos where 3000 ml of blood was drained [7]. The challenges associated with hematocolpos drainage of this large volume should be kept in mind when such cases are dealt with. Firstly, evacuation of a large hematocolpos may result in serious complications [7]. Hence, patient should be prepared for both abdominal and perineal surgery beforehand [7]. A rectoabdominal examination should be performed prior to incision on the imperforate hymen in order to determine any associated hematometra or hematosalpinx [7]. In our case, hematometra and hematosalpinx were ruled out on MRI and only vagina was involved. If only vagina is involved, the uterus may be felt as a separate small mass on the dome of the distended vagina although in our case we could not appreciate the uterus clinically on examination until after drainage of hematocolpos [7]. Boggy masses if felt in adnexal regions may suggest hematosalpinx or hematometra [7]. A hematosalpinx may be torn away from its adhesions to the parietal pentoneum after decompression of the vagina, which may result in intra-peritoneal hemorrhage [7]. If adnexal involvement is suspected, a laparotomy should be performed prior to the incision of the hymen in order to ensure the proper drainage of the hematosalpinx or hematometra [7]. A stab wound is first made in the hymen so as to allow very slow drainage of retained menstrual blood and then the stab incision is extended in cruciate manner only after the vagina is drained empty [7]. There is some possibility of adhesion of hymenal tags which might lead to partial recurrence of the condition. Hence, some authorities suggest excision of the hymen [7]. Drainage of blood from the vagina should not be hastened by giving pressure in the suprapubic region of the abdomen [7]. 

## Conclusions

Although a rare condition, imperforate hymen is easily diagnosed. The most common presenting symptom is painful cryptomenorrhea. Young teenage girls presenting with amenorrhea and cyclical abdominal pain should arouse suspicion of this condition. Surgical hymenotomy should be performed to treat the condition. Drainage of large volumes of hematocolpos should be done only after taking into account all the possible complications.
